# Physical activity across the adult life course and incident dementia in the Framingham Heart Study

**DOI:** 10.1002/alz.084425

**Published:** 2025-01-09

**Authors:** Phillip H Hwang, Chenglin Lyu, Chunyu Liu, Karin Schon, Jesse Mez, Rhoda Au

**Affiliations:** ^1^ The Framingham Heart Study, Framingham, MA USA; ^2^ Boston University School of Public Heatlh, Boston, MA USA; ^3^ Boston University Chobanian & Avedisian School of Medicine, Boston, MA USA; ^4^ Framingham Heart Study, Framingham, MA USA; ^5^ Boston University, Boston, MA USA; ^6^ Boston University School of Medicine, Boston, MA USA; ^7^ Framingham Heart Study, Boston, MA USA; ^8^ Boston University Alzheimer’s Disease Research Center, Boston, MA USA; ^9^ Department of Neurology, Boston University Chobanian & Avedisian School of Medicine, Boston, MA USA; ^10^ Davos Alzheimer’s Collaborative, Philadelphia, PA USA; ^11^ Slone Epidemiology Center, Boston University Chobanian & Avedisian School of Medicine, Boston, MA USA; ^12^ Department of Epidemiology, Boston University School of Public Health, Boston, MA USA; ^13^ Boston University Chobanian & Avedisian School of Medicine and School of Public Health, Boston, MA USA; ^14^ Department of Anatomy and Neurobiology, Neurology and Medicine, Framingham Heart Study, BU Alzheimer’s Disease Research Center, Boston University Chobanian & Avedisian School of Medicine, Boston, MA USA

## Abstract

**Background:**

Greater levels of physical activity are associated with improved cognition and decreased risk for dementia, but it is not clear when the potential benefits of physical activity on brain health are most beneficial throughout the life course. We examined associations between overall physical activity and incident dementia among adults in early adult life, midlife and late‐life.

**Method:**

Participants from the Framingham Heart Study Offspring cohort were included. Those in the early adult life group (26‐44 years), midlife group (45‐65 years), and late‐life group (66‐80 years) were selected from Exam 2 (1979‐1983), Exam 4 (1987‐1991), and Exam 7 (1998‐2001), respectively, who were dementia‐free at their baseline exams. Overall daily physical activity was based on the physical activity index (PAI) and analyzed as quintiles (Q1‐Q5). Diagnosis of all‐cause dementia was adjudicated by a multi‐disciplinary consensus committee through 2020. Cox models were used to evaluate associations between higher PAI (Q2‐Q5) versus low PAI (Q1) among early adult life, midlife, and late‐life groups and incident dementia.

**Result:**

A total of 1,740 participants comprised the early adult life group (mean age = 37 years), 2,385 participants comprised the midlife group (mean age = 54 years), and 953 participants comprised the late‐life group (mean age = 71 years). Higher PAI was associated with decreased dementia risk as compared to low PAI among the midlife group (HR = 0.72; 95% CI = 0.54 – 0.94; p = 0.02) and late‐life group (HR = 0.63; 95% CI = 0.45 – 0.87; p = 0.005), but not among the early adult life group (HR = 1.00; 95% CI = 0.53 – 2.26; p = 0.81).

**Conclusion:**

Greater levels of overall physical activity are significantly associated with reduced risk of dementia in midlife and late‐life adults. This suggests that having a physically active lifestyle earlier in life may be beneficial to brain health, though the impact of physical activity early in life and in early adulthood warrants further investigation.

